# Enhanced Reduction of Nitrate to Ammonia at the Co-N Heteroatomic Interface in MOF-Derived Porous Carbon

**DOI:** 10.3390/ma18132976

**Published:** 2025-06-23

**Authors:** Jing Liu, Shuo Du, Zibin Huang, Ning Liu, Zhichao Shao, Na Qin, Yanjie Wang, Hongfang Wang, Zhihui Ni, Liping Yang

**Affiliations:** 1 Center for Advanced Materials Research, Zhongyuan University of Technology, Zhengzhou 450007, China; 2 Laboratory of Environmental Sciences and Technology, Xinjiang Technical Institute of Physics & Chemistry, Chinese Academy of Sciences, Urumqi 830011, China

**Keywords:** electrocatalysis, NH_3_, nitrate reduction reaction, Co, MOF

## Abstract

The electrocatalytic reduction of nitrate is an efficient and green method for NH_3_ production. In this study, a Co-containing MOF with a stable three-dimensional carbon framework that offers abundant metal active sites is prepared as a precursor to a Co-N-C electrocatalyst. Facile pyrolysis of the three-dimensional MOF affords the desired Co-N-C electrocatalyst, which exhibits excellent stability, an NH_3_ yield of 1.12 mmol h^−1^ mg^−1^, and faradaic efficiency of 86.7% at −0.23 V in a 0.1 M KOH/0.1 M KNO_3_. The excellent activity and durability are ascribed to the highly exposed active centres, large surface area, and high porosity structure. N doping allows the electronic properties to be modulated and provides outstanding stability owing to the strong interaction between the nitrogen-doped carbon support and Co nanoparticles. This study presents a simple and efficient synthesis strategy for the production of non-noble-metal electrocatalysts with abundant active sites for the nitrate reduction reaction.

## 1. Introduction

NH_3_ is extensively used in various fields, including dyes, medicine, plastics, and fertiliser production [1], and is also recognised as a reproducible non-carbon energy carrier (4.32 kW h kg^−1^) [2]. Consequently, NH_3_ is among the most intensively produced chemicals worldwide. In industry, NH_3_ is mainly synthesised using the high-temperature/pressure Haber Bosch method, an energy-intensive process noted for its substantial carbon dioxide emissions. Accordingly, the production of NH_3_ via electrocatalytic nitrogen reduction has attracted considerable attention [3,4,5]. However, the process suffers from sluggish kinetics and low selectivity caused by the high N≡N energy barriers, poor solubility of nitrogen in water, and intense competition with hydrogen evolution [6]. The production of NH_3_ from nitrate reduction reaction (NO_3_RR) rather than from nitrogen is therefore advantageous owing to the lower N=O bond energy and outstanding water solubility of nitrate [7]. These advantageous properties enhance the reaction kinetics to enable the efficient synthesis of NH_3_. Nitrates are commonly found as contaminants in agricultural and industrial wastewater. The accumulation of nitrate has resulted in global acidification and the eutrophication of terrestrial ecosystems [8]. Accordingly, the reduction of nitrate not only mitigates pollution but is also useful to produce high-value NH_3_. NO_3_RR is a complex reaction involving an 8e^−^ transfer process and its conversion efficiency is limited due to various byproducts (including NO_2_^−^ and N_2_H_4_) [9,10]. Therefore, it is essential to synthesise NO_3_RR catalysts with advanced activity and selectivity to achieve the efficient reduction of nitrate to NH_3_.

Noble-metal-based catalysts have high nitrate reduction reaction (NO_3_RR) activity. However, their application is limited by their high cost [11,12,13]. Catalysts based on non-noble transition metals have therefore received significant attention as more economical alternatives [14,15,16]. In particular, Co-based catalysts are known to efficiently catalyse the NO_3_RR toward the synthesis of NH_3_. In addition, Co-based catalysts are inert toward the hydrogen evolution reaction (HER), resulting in highly selective conversion of nitrate to NH_3_ via the NO_3_RR [17,18]. Many Co-based compounds have been used to catalyse the NO_3_RR, including CoP [19,20], CoO_x_ [21,22], Co-N_x_ [23], and Co alloys [24,25]. However, these catalysts commonly exhibit poor conductivity and low stability, have few reactive sites, and are thus unable to simultaneously achieve a high yield and selectivity [26]. Attaining this goal would necessitate the design of electrocatalysts with abundant reactive sites and excellent electrical conductivity. In this regard, MOF-derived materials are promising electrocatalysts for the NO_3_RR owing to the alleviative HER, regulable microstructures, and large surface area [27,28,29]. Nitrogen-doped porous carbons derived from MOFs have been explored to improve NO_3_^−^ adsorption and activation ability for promoting the NO_3_RR kinetics [30]. For example, a Co/N-C material derived from the zeolitic imidazolate framework has abundant reactive sites and excellent electrical conductivity and is a highly suitable electrocatalyst for the production of NH_3_ from nitrate [31,32]. However, current research cannot meet the requirements of practical applications. Hence, developing more efficient NO_3_RR catalysts derived from MOF and improving the electrochemical NO_3_RR performance are necessary to investigate.

In this study, the Co-containing MOF (named CoCP), which has a stable 3D carbon framework and offers a large number of metal active sites, was prepared as a precursor to the Co-N-C catalyst, which was synthesised via the facile pyrolysis of CoCP. The N-rich ligand 4,4′-bpy was used to prepare the MOF precursor through a solvothermal reaction. N doping enables the modulation of the electronic properties. The obtained Co-N-C showed a porous architecture, high surface area, and high nitrogen content. Based on these appealing properties, Co-N-C exhibited excellent electrocatalytic activities and outstanding long-term durability.

## 2. Experimental Procedure

Synthesis of Co-N-C: The Co-contained MOF {[Co_3_(L)_2_(4,4′-bpy)_2_(H_2_O)_2_]∙14H_2_O}n (named CoCP) was selected as an ideal precursor for the development of Co-N-C through a pyrolysis route. Firstly, the precursor CoCP was prepared based on our previous findings [33]. Then, CoCP was heated to 800 °C under a nitrogen atmosphere for 1 h. CoCP successfully transformed the original 3D organometallic framework structure into cobalt metal nanoparticles based on nitrogen-doped carbon (Co-N-C) through the pyrolysis process.

## 3. Results and Discussion

**Scheme 1:**
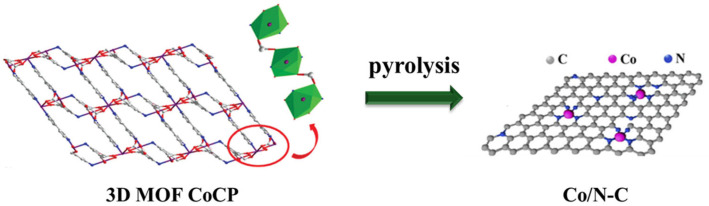
Illustration of the preparation of Co-N-C.

The Co-containing MOF CoCP, prepared using the procedure reported in our previous work [33], has a 3D structure (Figure S1), abundance of Co metal centres, and atomic level dispersion. The experimental X-ray diffraction (XRD) pattern is in good agreement with the simulated pattern (Figure S2 and Table S1), indicating the phase purity of CoCP crystalline. The Co-N-C composite was then synthesised by subjecting the Co-containing 3D MOF to pyrolysis (Scheme 1). The pyrolysis of CoCP generated homogeneously dispersed Co nanoparticles embedded in N-C support. The Co content in Co-N-C was 23.54 wt%, as determined by the inductively coupled plasma–atomic emission spectroscopy.

**Figure 1:**
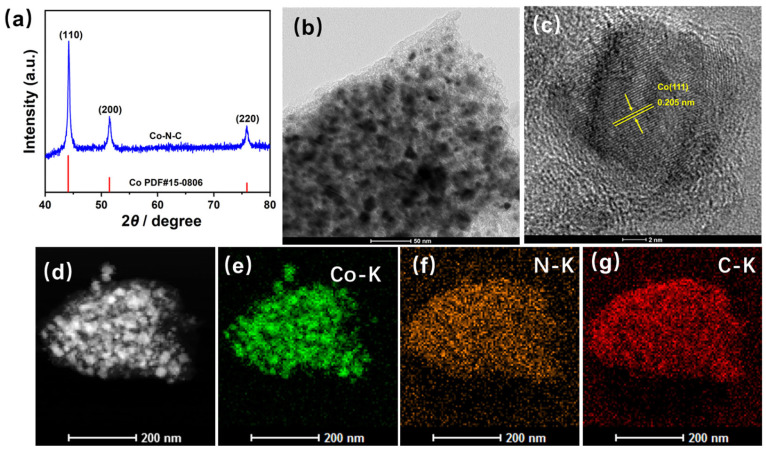
(**a**) XRD pattern, (**b**) TEM, (**c**) HR-TEM, and (**d**–**g**) EDX images of Co-N-C.

The XRD pattern confirmed the growth of highly crystalline Co nanoparticles on the N-C substrate (Figure 1a). The diffraction peaks located at 75.9°, 51.5°, and 44.2° were attributed to the 220, 200, and 110 planes of Co (PDF # 15-0806), respectively. Transmission electron microscopy (TEM) was adopted to display the structure of Co-N-C. Figure 1b reveals the homogeneous dispersion of Co nanoparticles embedded in the N-C support. Figure 1c exhibits the corresponding high-resolution TEM (HR-TEM) image, which enabled the lattice fringe to be determined as 0.204 nm, assigned to the (111) plane of Co (PDF # 15-0806) [34], consistent with the XRD results. In addition, energy-dispersive X-ray (EDX) mapping images and spectra (Figure 1d–g and Figure S3) further proved that Co, N, and C were uniformly distributed on the N-C support.

**Figure 2:**
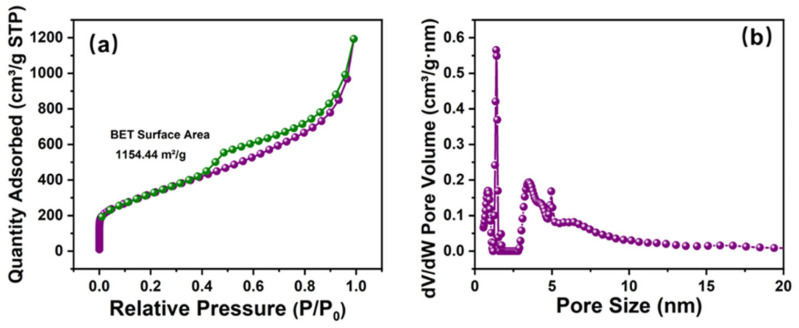
(**a**) N_2_ adsorption and desorption isotherms of Co-N-C (purple: adsorption isotherm and green: desorption isotherm) and (**b**) corresponding pore size distribution.

The efficiency of catalytic reactions is often highly influenced by the surface area of electrocatalysts. Accordingly, the surface area was characterised using nitrogen absorption–desorption isotherms (Figure 2a). The Co-N-C isotherms exhibit type IV behaviour, and the surface area of Co-N-C was 1154.44 m^2^ g^−1^, superior to that of many reported NO_3_RR catalysts (Table S2). Figure 2b reveals that Co-N-C has a micro-/mesoporous structure. The large surface area together with the porous structure facilitated charge and mass transport during electrocatalysis.

**Figure 3:**
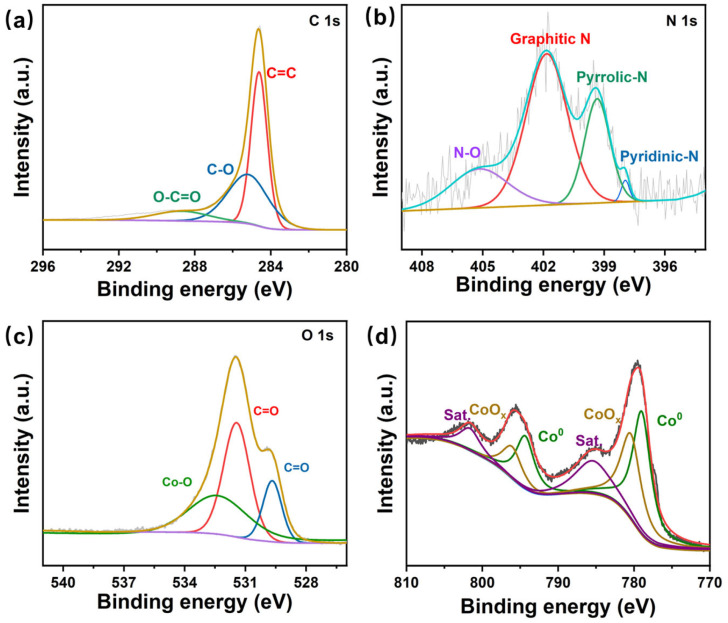
(**a**) C 1 s, (**b**) N 1 s, (**c**) O 1 s, and (**d**) Co 2p XPS spectra in Co-N-C.

Figure 3 shows the X-ray photoelectron spectroscopic (XPS) data used to illustrate the valence states of Co-N-C. The C 1 s peak at the position of 284.6 eV was employed as a reference to correct the binding energy. The survey spectrum proved the presence of C, N, Co, and O in the electrocatalyst Co-N-C (Figure S4). The peaks of C 1 s at ~284.6 eV, ~285.3 eV, and ~288.9 eV were attributed to C-C, C-O, and O-C=O bonds (Figure 3a), respectively [35]. The N 1 s spectra revealed four peaks at 397.9, 399.5, 401.8, and 405.2 eV, belonging to the pyridinic N, pyrrolic N, graphitic N, and N-O (Figure 3b), respectively [36]. In the O 1 s spectra (Figure 3c), the peaks at 529.7 eV and 531.4 eV are characteristic of C=O species. The additional peak at 532.5 eV was ascribed to the coordination between Co and O [37]. Figure 3d shows the Co 2p spectra. The major peaks at 778.8 eV and 794.4 eV were ascribed to the metallic Co, while the peaks at 780.5 eV and 796.3 eV were attributed to the CoO_x_ phase, which can arise from the oxidation of metallic Co [38]. The peaks at 785.5 eV and 801.8 eV were satellite peaks attributed to the shakeup excitation of Co^2+^ ions.

**Figure 4:**
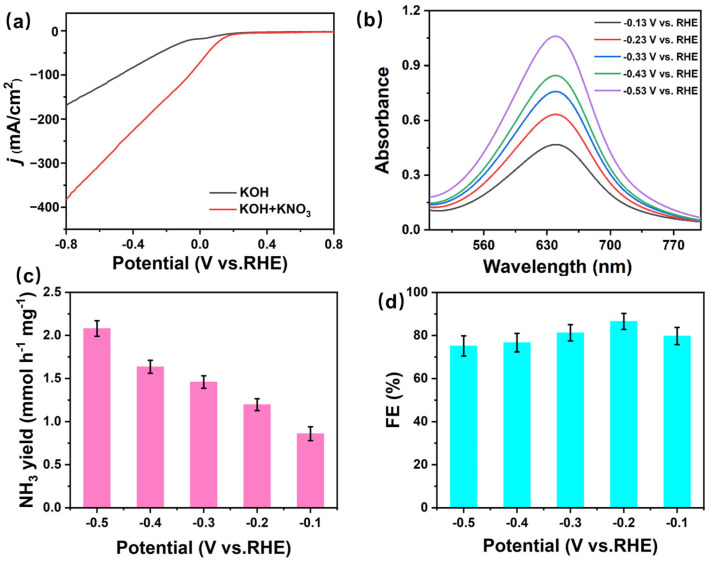
(**a**) LSV of Co-N-C in 0.1 M KOH and 0.1 M KOH/0.1 M KNO_3_ solutions. (**b**) UV-Vis spectra of Co-N-C for the NO_3_RR at different potentials (the obtained reaction solutions were diluted 6 times). (**c**) NH_3_ yield and (**d**) FE of Co-N-C at different potentials.

The NO_3_RR performance of Co-N-C was assessed in an H-type electrolytic cell. The production of NH_3_ was quantified using a colorimetric method. The linear sweep voltammetry (LSV) of Co-N-C revealed a remarkable increase in the current density from −0.8 to 0 V (vs. RHE) for the nitrate-containing electrolyte, indicating the high NO_3_RR efficiency of Co-N-C (Figure 4a). The NH_3_ concentration was calibrated by plotting the corresponding standard curve (Figure S5). The results showed that Co-N-C exhibited exceptional NO_3_RR performance with a high NH_3_ yield (1.12 mmol h^−1^ mg^−1^) and FE (86.7%) at −0.23 V in an electrolyte consisting of 0.1 M KOH/0.1 M KNO_3_. This performance is superior to that of many reported NO_3_RR catalysts (Table S3). The decreasing FE with increasing applied potential was attributed to competition between the NO_3_RR and the HER.

**Figure 5:**
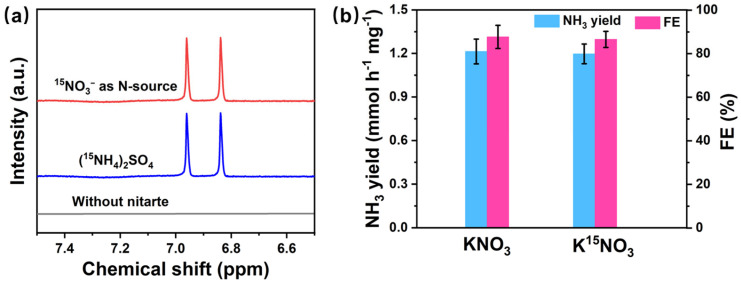
(**a**) 1H NMR spectra of (^15^NH_4_)_2_SO_4_ and Co-N-C using ^15^NO_3_^−^ as a nitrogen source for the NO_3_RR. (**b**) NH_3_ yield and FE of Co-N-C at −0.23 V in 0.1 M KOH/0.1 M KNO_3_ and 0.1 M KOH/0.1 M K^15^NO_3_, respectively.

An isotope-labelling electrocatalysis experiment in 0.1 M K^15^NO_3_ as the electrolyte was performed to confirm that the produced NH_3_ indeed originates from NO_3_RR. A blank experiment was conducted in the KOH electrolyte without nitrate; as shown in Figure 5a, no ammonia was detected. Peaks corresponding to ^15^N-labelled NH_3_ appeared in the 1H nuclear magnetic resonance (NMR) spectra in the 0.1 M KOH/0.1 M K^15^NO_3_. The concentration of the as-produced ^15^NH_3_ was consistent with the 14NH_3_ results at −0.23 V (Figure 5b), confirming that the N in NH_3_ does in fact come from the nitrate in the 0.1 M KNO_3_.

**Figure 6:**
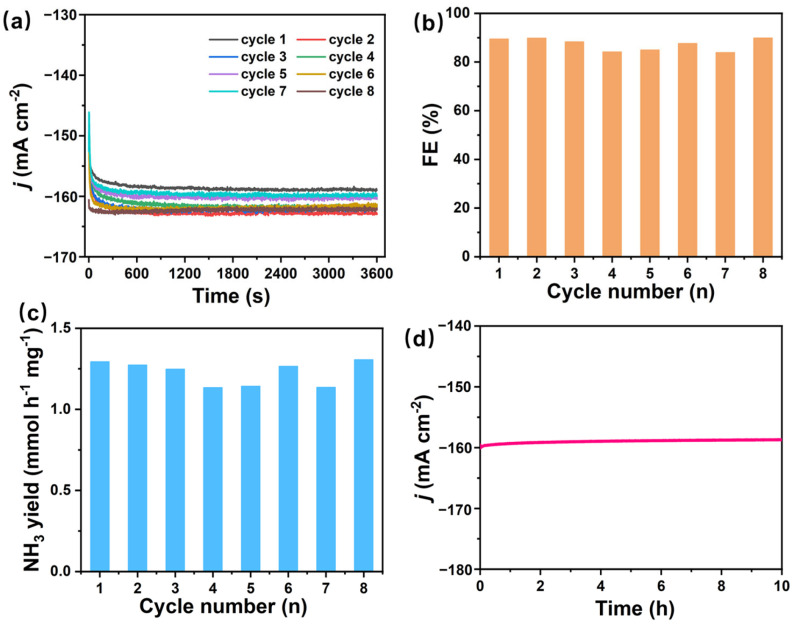
(**a**) j−t curves of Co-N-C in 0.1 M KOH/0.1 M KNO_3_. (**b**) NH_3_ yield rate and (**c**) FE for recycling tests over Co-N-C. (**d**) j−t curve for 10 h durability test.

**Figure 7:**
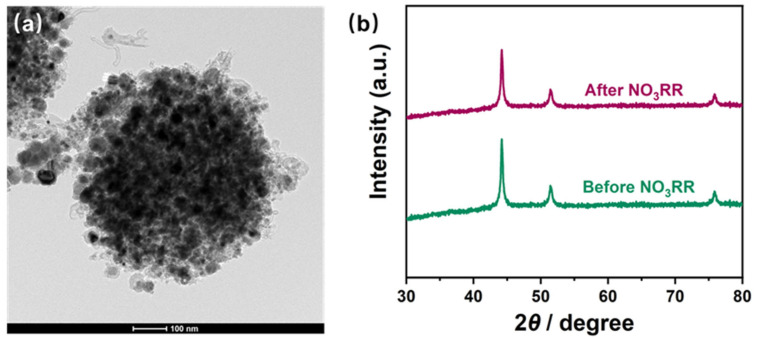
(**a**) TEM image and (**b**) XRD pattern of Co-N-C after 8 recycles NO_3_RR.

A durability assessment of Co-N-C indicated that the NH_3_ yield and FE did not decrease appreciably during eight recycling tests, thereby demonstrating the excellent electrocatalytic stability of Co-N-C (Figure 6a–c and the corresponding UV-Vis spectra shown in Figure S6). Additionally, the Co-N-C exhibited perfect durability during a 10 h chronoamperometry test at −0.23 V. As displayed by the chronoamperometric curve, Co-N-C underwent no observable degradation for the duration of the measurement, demonstrating the excellent stability of the catalyst (Figure 6d). Additionally, the structure and morphology of Co-N-C measured after the durability tests were identical to those measured before the NO_3_RR (Figure 7), further confirming the high structural stability. Based on the above results, we concluded that Co-N-C possesses excellent stability and that this could be ascribed to the strong interactions between the Co nanoparticles and the N-C support, which suppress the aggregation, migration, and growth of the Co nanoparticles.

## 4. Conclusions

Nitrate was electrochemically reduced to NH_3_ using a Co-N-C catalyst derived from a 3D MOF to afford a high NH_3_ yield rate (1.12 mmol h^−1^ mg^−1^) and FE (86.7%) at −0.23 V in a 0.1 M KOH/0.1 M KNO_3_ solution. The enhanced NO_3_RR performance and excellent stability were achieved owing to a large number of highly exposed active centres, high porosity structure, and large specific surface area. N doping enables the modulation of the electronic properties, while the strong interaction between the Co nanoparticles and N-C substrate endows outstanding stability. The findings of the present work are expected to contribute to the design of advanced NO_3_RR catalysts. 

## Data Availability

The original contributions presented in this study are included in the article. Further inquiries can be directed to the corresponding author.

## Supplementary Materials

Experimental Section: The following supporting information can be downloaded at: https://www.mdpi.com/article/10.3390/ma18132976/s1, Figure S1: View of CoCP. Figure S2: XRD patterns of Co-MOF precursor. Figure S3: EDX spectra of Co-N-C. Figure S4: Survey XPS spectra. Figure S5: (a) The UV-Vis spectra and (b) the corresponding calibration curve. Figure S6: UV-Vis spectra of Co-N-C for the NO_3_RR during durability tests (the obtained reaction solutions were diluted 6 times). Table S1: Crystallographic data and structure refinement for Co-MOF precursor. Table S2: Comparison of the surface area of reported electrocatalysts and Co-N-C. Table S3. Comparison of NO RR performances of Co-N-C and reported electrocatalysts. References [11,12,20,23,34,39,40,41,42,43,44,45,46,47,48,49,50,51,52,53,54,55,56].
