# Supplementary material for: Enhanced Reduction of Nitrate to Ammonia at the Co-N Heteroatomic Interface in MOF-Derived Porous Carbon
Source: Materials (Basel). 2025 Jun 23;18(13):2976. doi: 10.3390/ma18132976 (PMC12250854; doi:10.3390/ma18132976)
Supplement: Supplementary file 1 [file materials-18-02976-s001.zip › materials-3659814-supplementary.pdf]

Supplementary Information

Enhanced Reduction of Nitrate to Ammonia at the Co-N Heteroatomic Interface in MOF-Derived Porous Carbon

Jing Liu [1,*], Shuo Du [1], Zibin Huang [1], Ning Liu [1], Zhichao Shao [1], Na Qin [1], Yanjie Wang [1], Hongfang Wang [1], Zhihui Ni [1] and Liping Yang [2,*]

[1] Center for Advanced Materials Research, Zhongyuan University of Technology, Zhengzhou 450007, China
[2] Laboratory of Environmental Sciences and Technology, Xinjiang Technical Institute of Physics & Chemistry, Chinese Academy of Sciences, Urumqi 830011, China
* Correspondence: 6784@zut.edu.cn (J.L.); yanglp@ms.xjb.ac.cn (L.Y.)

Experimental Section

Materials

Potassium hydroxide, potassium nitrate, salicylic acid, ammonium chloride, sodium hydroxide, and Absolute ethyl alcohol were purchased from Innochem Technology Co., Ltd. Nafion solution, sodium hypochlorite, sodium nitroprusside dihydrate and Tricisodium citrate dihydrate were supplied by Aladdin Company. All chemicals were used as received without further purification.

Electrochemical Measurements

Electrochemical experiments were performed at room temperature on an Zennium-pro electrochemical workstation in an H-type electrolytic cell separated by a Nafion 117 membrane under ambient temperature and standard atmospheric pressure. Pt foil electrode and Ag/AgCl electrode served as counter electrode and reference electrode, respectively. The preparation of the working electrode was as follows: catalysts (10 mg), Nafion (25 μL, 5 wt %) and ethyl alcohol (475 μL) were mixed and sonicated for 30 min. This process was used to create catalyst inks.10 μL of the suspension was casted on a glassy carbon disk(5 mm in diameter) as the working electrode and then dried in air. 0.1 M KOH solution (25 mL) was evenly distributed in the cathode and anode compartments. All experiments were performed in 0.1 M KOH with 0.1 M KNO_3 solution. All potentials are reported versus reversible hydrogen electrode (RHE) via converting the measured potentials using Equation (S1):

$$E_{RHE} = E_{Ag/AgCl} + 0.059 \times pH + 0.197 \tag{S1}$$

Calculation of NO_3RR Performance:

Equations of cathode reaction of NO_3RR:

$$NO_3^- + 8e^- + 7\ H_2O \rightarrow NH_4 + 10\ OH^- \quad (S2)$$

FE toward NH_3 via NO_3^- reduction reaction was calculated by equation:

$$FE=8 \times F \times [NH_4^+] \times V / M_{NH_4^+} \times Q \times 100\% \quad (S3)$$

NH_3 yield rate was calculated using the following equation:

$$NH_3 \text{ yield rate}=[NH_4^-] \times V / (M_{NH_4^+} \times t \times m_{cat.}) \quad (S4)$$

Where F is the Faradic constant (96485 C mol^{-1}), [NH_3] is the measured NH_3 concentration, V is the volume of electrolyte in the anode compartment (25 mL), M_{NH4^+} is the molar mass of N NH_4^+, Q is the total quantity of applied electricity; t is the electrolysis time and $m_{cat.}$ is the loaded mass of catalyst.

^{15}N Isotope Labeling Experiments

^{15}N isotopic labeling experiments were conducted using 0.1 M KOH with 0.1 M $K^{15}NO_3$ solution as the electrolyte with the same experimental procedure of 0.1 M KOH with 0.1 M KNO_3 experiments. The yielded $^{15}NH_3$ was measured by the 1H NMR methods with D_2O used as an internal standard using Bruker Avance-400 MHz.

Characterization Technique.

The X-ray diffraction (XRD) pattern was acquired on a Bruker D8 diffractometer. Transmission electron microscope (TEM) images and high-angle annular dark-field scanning transmission electron microscope (HAADF-STEM) images were conducted on a FEI f20 microscope. Energy dispersive X-ray spectroscopy (EDS) was measured on the EDS8000. X-ray photoelectron spectroscopy (XPS) was obtained on an AXISHSi spectrometer. Brunauer-Emmett-Teller (BET) surface area was obtained on Micro-meritics ASAP2020. Nuclear magnetic resonance (NMR) spectra are collected by a Bruker AVANCE 600 MHz nuclear magnetic resonance instrument.

Determination of NH_3

Concentration of produced NH_3 was determined by spectrophotometry measurement with indophenol blue method [1]. In detail, 2 mL of the diluted catholyte was obatined from the cathodic chamber and mixed with 2 mL of a 1 M NaOH solution that contained salicylic acid and sodium citrate. Then, 1 mL of 0.05 M NaClO and 0.2 mL of 1 wt% $C_5FeN_6Na_2O$ were dropped in the collected electrolyte solution. After standing at room temperature for 1 h, the ultraviolet-visible absorption spectrum was measured. The concentration-absorbance curve was calibrated using the standard NH_4Cl solution with NH_3 concentrations of 0, 0.4, 0.8, 1.2, 1.6, 2.0, 2.4, and 4.0 μg mL^{-1} in 0.1 M KOH. The absorbance at 655 nm was measured to quantify the NH_3 concentration using standard NH_4Cl solutions (y=0.3569x+0.0055, R^2 = 0.999).

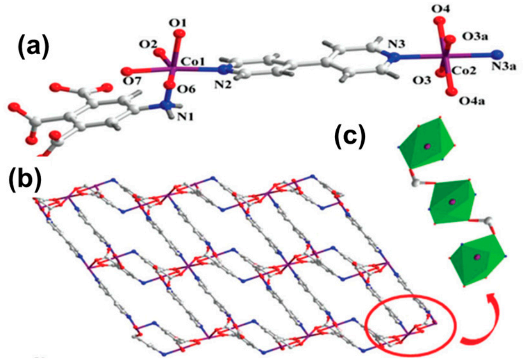

Figure S1. (a) View of the coordination environment of Co^{2+} ions. (b) View of the three nuclear units in CoCP. (c) View of the 3D network.

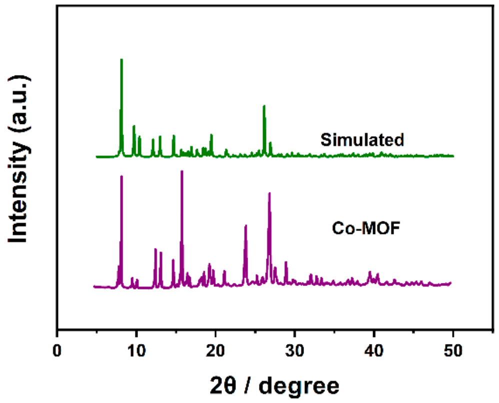

Figure S2. XRD patterns of Co-MOF precursor.

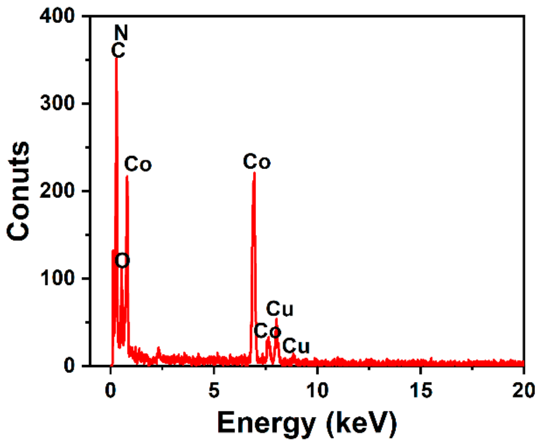

Figure S3. EDX spectra of Co-N-C.

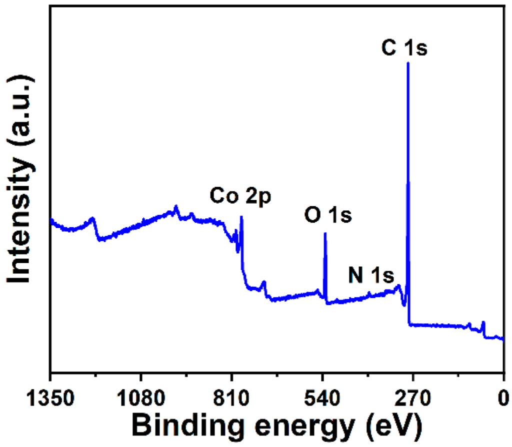

Figure S4. Survey XPS spectra and high resolution XPS spectra.

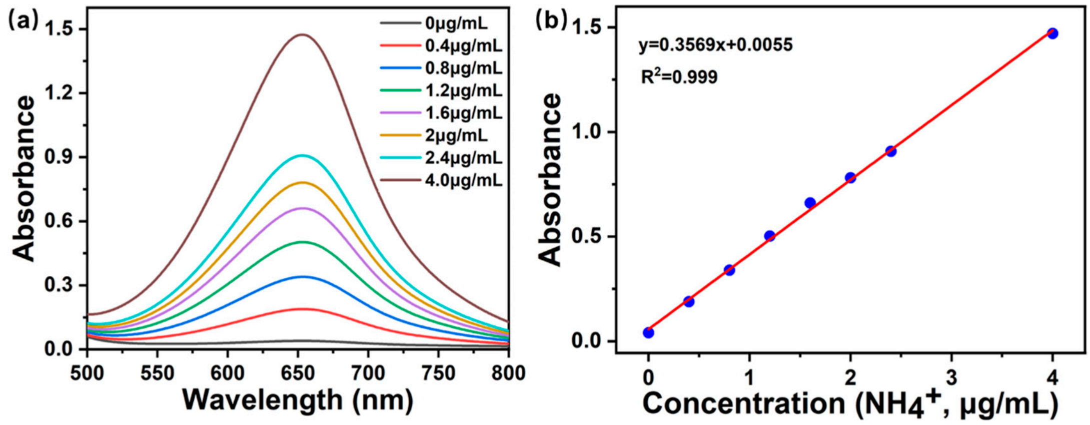

Figure S5. (a) The UV-Vis absorption spectra and (b) the corresponding calibration curve of the colorimetric NH_4^+ assay in 1 M KOH using the indophenol blue method.

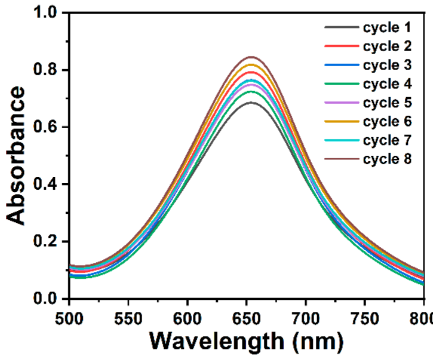

Figure S6. UV-Vis spectra of Co-N-C for the NO_3RR during recycling tests at − 0.23 V vs. RHE (the obtained reaction solutions were diluted 6 times).

Table S1. Crystallographic data and structure refinement for Co-MOF precursor.

Compound	Co-MOF Precursor
formula	$C_{38}H_{28}Co_3N_6O_{14}$
F_w	969.45
T/K	293(2)
l (Mo–Ka)/Å	0.71073
Crystal system	*Monoclinic*
Space group	*C2/c*
a/Å	28.7693(11)
b/Å	10.0680(4)
c/Å	21.9942(9)
α (deg)	90
β (deg)	128.3810(10)
γ (deg)	90
V ($Å^3$)	4993.9(3)
Z	4
D_{calcd}(g cm^{-3})	1.289
F(000)	1964
μ (mm^{-1})	1.046
GOF	0.993
R_1 ($I > 2\sigma(I)$)	0.0320
wR_2 ($I > 2\sigma(I)$)	0.0973

Table S2. Comparation the surface area of Co-N-C and reported electrocatalysts.

Catalyst	Surface Area/ m^2 g^{-1}	Reference
Co-N-C	1154.44	This work
Fe/Cu-HNG	858	[2]
RuFe-FeNC	397.34	[3]
ISAS-Fe/NC	389.6	[4]
NPC950	1084.5	[5]
NC@Ru	822.05	[6]
Co-Fe@Fe_2O_3.	350.12	[7]
NH_2-Zn-MOF	900	[8]
$NHPC_{1:3}$-900	1302	[9]
NPC-900	279.5	[10]
e-MOF-808	1407	[11]

Table S3. Comparation NO_3RR performances of Co-N-C and reported electrocatalysts.

Catalyst	Electrolyte	NH_3 yield rate (V vs. RHE)	FE (%) (V vs. RHE)	Refs.
Co-N-C	0.1 M KOH ($0.1 M KNO_3$)	1.12 mmol h^{-1} g^{-1} V= − 0.23	86.7 V= − 0.23	This work
CoPc-RGO	0.1 M K_2SO_4 (200 ppm KNO_3)	58.82 μg h^{-1} mg^{-1} V= − 0.2	95.12 V= − 0.2	[12]
2.5% Ag-Co_3O_4	0.1 M KOH (0.1 M KNO_3)	52 μmol h^{-1} cm^{-2} V= − 3.2	88 V= − 3.2	[13]
In-S-G	1 M KOH (0.1 M KNO_3)	0.22 mmol h^{-1} mg^{-1} V= − 0.7	75 V= − 0.5	[14]
PTCDA/O-Cu	0.1 M PBS (500 ppm NO_3^-)	0.436 mg h^{-1} cm^{-2} V= − 0.4	85.9 V= − 0.4	[15]
Fe SAC	0.10 M K_2SO_4 (0.5 M NO_3^-)	7.82 mg h^{-1} cm^{-2} V= − 0.85	75 V= − 0.66	[16]
Fe/Cu-NG	1.0 M KOH (0.10 M KNO_3)	1.08mmol h^{-1} mg^{-1} V= − 0.3	92.51 V= − 0.3	[17]
Pd/NF	0.5 M Na_2SO_4 (0.1 M $NaNO_3$)	1.52 mmol h^{-1} cm^{-2} V= − 1.0	80 V= − 1.0	[18]
Cu nanodisks	0.1 M KOH (10 mM KNO_3)	2.16 mg h^{-1} mg^{-1} V= − 0.63	81.11 V= − 0.63	[19]
PdCoO/NF	0.5 M K_2SO_4 (200 ppm $NaNO_3$)	0.204 mmol h^{-1} cm^{-2} V= − 1.3	88.6 V= − 1.3	[20]
np-CuCo	1M KOH (600 ppm NO_3^-)	578.7 μmol h-1 cm^{-2} V= − 0.23	93.8 V= − 0.23	[21]
TiO_{2-x}	0.5 M Na_2SO_4 (50 ppm NO_3^-)	0.765 mg h^{-1} cm^{-2} V= − 0.95	85 V= − 0.95	[22]
Co_1-P/NPG	0.5 M K_2SO_4 (0.1 M KNO_3)	21.7 mg h^{-1} mg^{-1} V= − 0.9	93.8 V= − 0.7	[23]
PP-Co/CP	0.1 M NaOH (0.1 M $NaNO_3$)	1.1 mmol h^{-1} mg^{-1} V= − 0.6	90.1 V= − 0.6	[24]

References

1. Zhu, D.; Zhang, L.; Ruther, R.E.; Hamers, R.J. Photo-illuminated diamond as a solid-state source of solvated electrons in water for nitrogen reduction. *Nat. Mater.* **2013**, *12*, 836–841.
2. Zhang, S.; Wu, J.; Zheng, M.; Jin, X.; Shen, Z.; Li, Z.; Wang, Y.; Wang, Q.; Wang, X.; Wei, H.; Zhang, J.; Wang, P.; Zhang, S.; Yu, L.; Dong, L.; Zhu, Q.; Zhang, H.; Lu, J. Fe/Cu diatomic catalysts for electrochemical nitrate reduction to ammonia. *Nat. Commun.* **2023**, *12*, 3634.
3. Zhao, X.; Jiang, Y.; Wang, M.; Liu, S.; Wang, Z.; Qian, T.; Yan, C. Optimizing Intermediate Adsorption via Heteroatom Ensemble Effect over RuFe Bimetallic Alloy for Enhanced Nitrate Electroreduction to Ammonia. *Adv. Energy Mater.* **2023**, *13*, 2301409.
4. Lü, F.; Zhao, S.; Guo, R.; He, J.; Peng, X.; Bao, H.; Fu, J.; Han, L.; Qi, G.; Luo, J.; Tang, X.; Liu, X. Nitrogen-coordinated single Fe sites for efficient electrocatalytic N2 fixation in neutral media. *Nano Energy* **2019**, *61*, 420–427.
5. Liu, Y.; Su, Y.; Quan, X.; Fan, X.; Chen, S.; Yu, H.; Zhao, H.; Zhang, Y.; Zhao, J. Facile Ammonia Synthesis from Electrocatalytic N2 Reduction under Ambient Conditions on N-Doped Porous Carbon. *ACS Catal.* **2023**, *13*, 16286.
6. Zhang, Z.; Yao, K.; Cong, L.; Yu, Z.; Qu, L.; Huang, W. Facile synthesis of a Ru-dispersed N-doped carbon framework catalyst for electrochemical nitrogen reduction. *Catal. Sci. Technol.* **2020**, *10*, 1336–1342.
7. Zhang, S.; Li, M.; Li, J.; Song, Q.; Liu, X. High-ammonia selective metal–organic framework–derived Co-doped Fe/Fe_2O_3 catalysts for electrochemical nitrate reduction. *Proc. Natl. Acad. Sci. USA* **2022**, *119*, e2115504119.
8. Wang, H.; Wu, X.; Liu, G.; Wu, S.; Xu, R. Bimetallic MOF derived nickel nanoclusters supported by nitrogen-doped carbon for efficient electrocatalytic CO_2 reduction. *Nano Res.* **2022**, *16*, 4546–4553.

9. Xuan, C.; Hou, B.; Xia, W.; Peng, Z.; Shen, T.; Xin, H.L.; Zhang, G.; Wang, D. From a ZIF-8 polyhedron to three-dimensional nitrogen doped hierarchical porous carbon: An efficient electrocatalyst for the oxygen reduction reaction. *J. Mater. Chem. A* **2018**, *6*, 10731–10739.
10. Wang, S.; Liu, L.; Wang, S.-M.; Han, Z. MOF-templated nitrogen-doped porous carbon materials as efficient electrocatalysts for oxygen reduction reactions. *Inorg. Chem. Front.* **2017**, *4*, 1231–1237.
11. Yang, S.-C.; Muthiah, B.; Chang, J.-W.; Tsai, M.-D.; Wang, Y.-C.; Li, Y.-P.; Kung, C.-W. Support effect in metal–organic framework-derived copper-based electrocatalysts facilitating the reduction of nitrate to ammonia. *Electrochim. Acta* **2024**, *492*, 144348.
12. Paul, S.; Sarkar, S.; Adalder, A.; Kapse, S.; Thapa, R.; Ghorai, U.K. Strengthening the Metal Center of Co–N_4 Active Sites in a 1D–2D Heterostructure for Nitrate and Nitrogen Reduction Reaction to Ammonia. *ACS Sustain. Chem. Eng.* **2023**, *11*, 6191–6200.
13. Zhang, M.; Ma, Z.; Zhou, S.; Han, C.; Kundi, V.; Kumar, P.V.; Thomsen, L.; Johannessen, B.; Peng, L.; Shan, Y.; et al. Surface Engineering on Ag-Decorated Co_3O_4 Electrocatalysts for Boosting Nitrate Reduction to Ammonia. *ACS Catal*. **2024**, *14*, 11231–11242.
14. Lei, F.; Xu, W.; Yu, J.; Li, K.; Xie, J.; Hao, P.; Cui, G.; Tang, B. Electrochemical synthesis of ammonia by nitrate reduction on indium incorporated in sulfur doped graphene. *Chem. Eng. J.* **2021**, *426*, 131317.
15. Chen, G.-F.; Yuan, Y.; Jiang, H.; Ren, S.-Y.; Ding, L.-X.; Ma, L.; Wu, T.; Lu, J.; Wang, H. Electrochemical reduction of nitrate to ammonia via direct eight-electron transfer using a copper–molecular solid catalyst. *Nat. Energy* **2020**, *5*, 605–613.
16. [16] Z.-Y. Wu, M. Karamad, X. Yong, Q. Huang, D.A. Cullen, P. Zhu, C. Xia, Q. Xiao, M. Shakouri, F.-Y. Chen, J.Y. Kim, Y. Xia, K. Heck, Y. Hu, M.S. Wong, Q. Li, I. Gates, S. Siahrostami, H. Wang, Electrochemical ammonia synthesis via nitrate reduction on Fe single atom catalyst, Nat. Commun., 12 (2021) 2870.
17. Wu, Z.-Y.; Karamad, M.; Yong, X.; Huang, Q.; Cullen, D.A.; Zhu, P.; Xia, C.; Xiao, Q.; Shakouri, M.; Chen, F.-Y.; Kim, J.Y.; Xia, Y.; Heck, K.; Hu, Y.; Wong, M.S.; Li, Q.; Gates, I.; Siahrostami, S.; Wang, H. Electrochemical ammonia synthesis via nitrate reduction on Fe single atom catalyst. *Nat. Commun.* **2021**, *12*, 2870.
18. Guo, H.; Li, M.; Yang, Y.; Luo, R.; Liu, W.; Zhang, F.; Tang, C.; Yang, G.; Zhou, Y. Self-Supported Pd Nanorod Arrays for High-Efficient Nitrate Electroreduction to Ammonia. *Small* **2023**, *19*, 2207743.
19. Wu, K.; Sun, C.; Wang, Z.; Song, Q.; Bai, X.; Yu, X.; Li, Q.; Wang, Z.; Zhang, H.; Zhang, J.; Tong, X.; Liang, Y.; Khosla, A.; Zhao, Z. Surface Reconstruction on Uniform Cu Nanodisks Boosted Electrochemical Nitrate Reduction to Ammonia. *ACS Mater. Lett.* **2022**, *4*, 650–656.
20. Liu, M.; Mao, Q.; Shi, K.; Wang, Z.; Xu, Y.; Li, X.; Wang, L.; Wang, H. Electroreduction of Nitrate to Ammonia on Palladium–Cobalt–Oxygen Nanowire Arrays. *ACS Appl. Mater. Interfaces* **2022**, *14*, 13169–13176.
21. Zhou, X.; Xu, W.; Liang, Y.; Jiang, H.; Li, Z.; Wu, S.; Gao, Z.; Cui, Z.; Zhu, S. Dynamically Restructuring Nanoporous Cu–Co Electrocatalyst for Efficient Nitrate Electroreduction to Ammonia. *ACS Catal.* **2024**, *14*, 12251–12259.
22. Jia, R.; Wang, Y.; Wang, C.; Ling, Y.; Yu, Y.; Zhang, B. Boosting Selective Nitrate Electroreduction to Ammonium by Constructing Oxygen Vacancies in TiO_2. *ACS Catal.* **2020**, *10*, 3533–3540.
23. Ni, J.; Yan, J.; Li, F.; Qi, H.; Xu, Q.; Su, C.; Sun, L.; Sun, H.; Ding, J.; Liu, B. Atomic Co–P Catalytic Pair Drives Efficient Electrochemical Nitrate Reduction to Ammonia. *Adv. Energy Mater.* **2024**, *14*, 2400065.
24. Chen, Q.; Liang, J.; Liu, Q.; Dong, K.; Yue, L.; Wei, P.; Luo, Y.; Liu, Q.; Li, N.; Tang, B.; et al. Co nanoparticle-decorated pomelo-peel-derived carbon enabled high-efficiency electrocatalytic nitrate reduction to ammonia. *Chem. Commun.* **2022**, *58*, 4259–4262.
